# The Quasi‐Binary Acetonitriletriide Sr_3_[C_2_N]_2_

**DOI:** 10.1002/anie.201912831

**Published:** 2019-11-19

**Authors:** William P. Clark, Andreas Köhn, Rainer Niewa

**Affiliations:** ^1^ Institut für Anorganische Chemie Universität Stuttgart Pfaffenwaldring 55 70569 Stuttgart Germany; ^2^ Institut für Theoretische Chemie Universität Stuttgart Pfaffenwaldring 55 70569 Stuttgart Germany

**Keywords:** acetonitriletriide, pseudonitride, Raman spectroscopy, solid-state synthesis, structure determination

## Abstract

The first quasi‐binary acetonitriletriide Sr_3_[C_2_N]_2_ has been synthesised and characterised. The nearly colourless crystals were obtained from the reaction of Sr metal, graphite, and elemental N_2_, generated by decomposition of Sr(N_3_)_2_, in a sealed Ni ampoule with the aid of an alkali metal flux. The structure of this compound was analysed via single‐crystal X‐ray diffraction and the identity of the [C_2_N]^3−^ anion was confirmed by Raman spectroscopy and further investigated by quantum‐chemical methods. Computed interatomic distances within the [C_2_N]^3−^ anion strikingly match the obtained experimental data.

## Graphical abstract

**Crystals** of the first quasi‐binary acetonitriletriide Sr_3_[C_2_N]_2_ were obtained from the solid‐state reaction of Sr metal, graphite, and Sr(N_3_)_2_. The identity of the nearly colourless crystals was proven via single‐crystal X‐ray diffraction and Raman spectroscopy. Quantum‐chemical calculations give deeper insights into bonding and charge distribution within the anion.

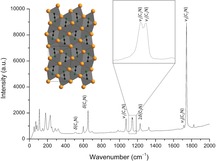

The fully deprotonated acetonitriletriide anion, [C_2_N]^3−^, is a linear three‐atom unit, which is isoelectronic to both molecular CO_2_ and the carbodiimide ion, [CN_2_]^2−^. The acetonitrile molecule, H_3_CCN, contains both a C≡N and C−C bond; however, when the molecule is deprotonated, the C–N bonding weakens while the C–C bonding gets stronger.^1^ This results in the fully deprotonated species, [C_2_N]^3−^, containing both C=N and C=C bonds, which feature strongly delocalised electron pairs. Similar to [CN_2_]^2−^, which may be considered a pseudooxide ion, [C_2_N]^3−^ can be viewed as a pseudonitride ion. This anion has been rarely observed, with the only solid‐state examples reported to date being Ba_5_[TaN_4_][C_2_N] and Sr_4_N[CN_2_][C_2_N].^1^, ^2^ The discovery that these anions could be stabilised in a solid‐state system lead to the question whether quasi‐binary acetonitriletriide compounds could be produced, since the similar anion [CN_2_]^2−^ produces a large range of quasi‐binary compounds, for example, *M*_2_[CN_2_] (*M*=Li–Cs)^3^, ^4^, ^5^, ^6^, ^7^ and *M*[CN_2_] (*M*=Be–Ba, Mn–Zn, Cd, Pb, and Eu).^8^, ^9^, ^10^, ^11^, ^12^, ^13^, ^14^, ^15^, ^16^, ^17^, ^18^ In addition, other linear three‐atom ions, such as [N_3_]^−^, [C_3_]^4−^, [BN_2_]^3−^, [OCN]^−^, and [CBN]^4−^, have also produced quasi‐binary and higher compounds.^19^, ^20^, ^21^, ^22^, ^23^, ^24^ Herein, we report the first quasi‐binary acetonitriletriide, which was characterised by single‐crystal X‐ray diffraction, Raman spectroscopy, and elemental analysis, as well as quantum‐chemical methods.

A single crystal of the title compound, a nearly colourless shard with a hint of green, was measured with a single‐crystal X‐ray diffractometer and indexed in a monoclinic unit cell (*a=*4.0745(1) Å, *b=*10.7254(5) Å, *c=*7.0254(3) Å, *β*=102.700(2)°). Refinement of the single‐crystal structural data showed that Sr_3_[C_2_N]_2_ crystallises in the space group *P*2_1_/*c* (No. 14) and is non‐merohedrally twinned (Tables S1–S3). The structure can be described as comprising face‐ and edge‐sharing bidisphenoid polyhedra of Sr, with one [C_2_N]^3−^ unit located inside each bidisphenoid (Figure 1). Such bidisphenoid polyhedra are also seen in Sr_4_N[CN_2_][C_2_N],^2^ which likewise contain Sr as the sole cation. This observation can be viewed as an indication that the acetonitriletriide anions prefer to be situated in bidisphenoid polyhedra of Sr cations. Each bidisphenoid is connected to 18 other bidisphenoids, of which 9 are edge‐sharing, 4 are face‐sharing, and 5 are corner‐sharing. The Sr cations have two crystallographically independent positions: one is coordinated by C and N atoms to form octahedra and the other position is coordinated by five C and N atoms to form a distorted trigonal bipyramid. Each octahedron is connected in total to 6 distorted trigonal bipyramids, 4 of which are corner‐sharing and 2 are edge‐sharing.

**Figure 1:**
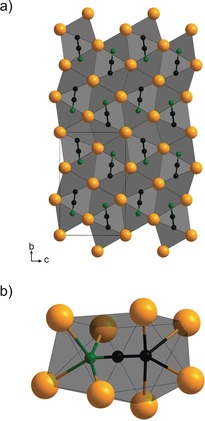
a) Section of the crystal structure of Sr_3_[C_2_N]_2_, viewed along the *a*‐axis; b) Bidisphenoid coordination environment of the [C_2_N]^3−^ anions. Sr: orange, C: black, and N: green.

The nearly linear [C_2_N]^3−^ units (177.9(6)°) have almost equal interatomic distances (1.278(8) Å and 1.288(9) Å), which do not fit with those typically seen for C=N and C=C bonds (1.23 Å and 1.35 Å, respectively).^25^, ^26^ Attempts to separately simulate both complete ordering of the [C_2_N]^3−^ units and alternating orientations of the units resulted in only marginal differences in the refinement results. Alternating orientations of the [C_2_N]^3−^ units, which could also be referred to as a disorder, have been previously reported for Ba_5_[TaN_4_][C_2_N].^1^ In this case an average of the interatomic distances would be seen and the resultant average interatomic distance of C=C/N from the aforementioned literature values, 1.29 Å, does fit well with what was observed in the structural data. Due to the limitation of standard X‐ray diffraction techniques, such alternating orientations of the [C_2_N]^3−^ units could not be unambiguously excluded; however, quantum‐chemical calculations indicate its absence (see below). Additionally, the distances between the coordinating strontium atoms and the shorter end of the complex ion, about 2.65 Å, perfectly match distances expected between nitrogen and strontium, while the significant longer distance to the longer end, around 2.80 Å, indicate carbon to strontium distances. Neutron diffraction would be the most appropriate method to confirm the orientations of the [C_2_N]^3−^ units; however, the synthesis of this compound is challenging and the compound itself is highly air‐sensitive and thus a larger quantity of sample could not be produced.

To confirm the identity of the [C_2_N]^3−^ units, Raman spectroscopy was employed. The obtained spectrum and vibrational frequencies (Figure 2 and Table 1) were compared with previously observed values for the [C_2_N]^3−^ unit. The frequencies observed for the symmetric (*ν*_1_=1146 cm^−1^) and asymmetric (*ν*_3_=1744 and 1826 cm^−1^) stretching, as well as the deformation vibration frequencies (*δ*=508–651 cm^−1^), fit well with what has been previously seen for [C_2_N]^3−^ in Ba_5_[TaN_4_][C_2_N] (*ν*_1_=1118 and 1127 cm^−1^, *ν*_3_=1724 and 1734 cm^−1^, and *δ*=460 and 503 cm^−1^)^1^ and Sr_4_N[C_2_N][CN_2_] (*ν*_1_=1152 cm^−1^, *ν*_3_=1767 and 1809 cm^−1^, and *δ*=615 and 715 cm^−1^).^2^

**Figure 2:**
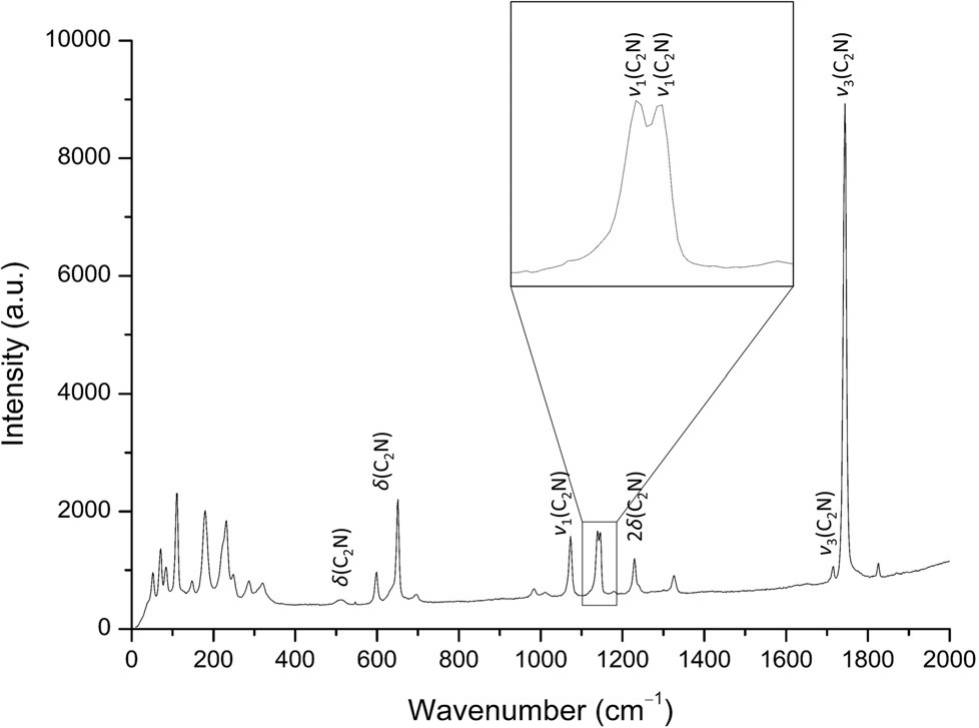
Raman spectrum of Sr_3_[C_2_N]_2_ measured using a green laser (*λ*=532 nm) along with assigned vibrational frequencies.

**Table 1:** Vibrational frequencies from the Raman spectrum of Sr_3_[C_2_N]_2_. Relative intensity is denoted by the following descriptions: very strong (vs) ≥90 %, weak (w) 30–10 % and very weak (vw) ≤10 %.

	Raman [cm^−1^]	Assignment	
	508 vw	*δ*(C_2_N)	
	599 vw	*δ*(C_2_N)	
	651 vw	*δ*(C_2_N)	
	1073 w	*ν*_1_(C_2_N)	
	1139 w	*ν*_1_(C_2_N)	
	1146 w	*ν*_1_(C_2_N)	
	1230 w	2*δ*(C_2_N)	
	1715 w	*ν*_3_(C_2_N)	
	1744 vs	*ν*_3_(C_2_N)	
	1826 w	*ν*_3_(C_2_N)

During the synthesis of Sr_3_[C_2_N]_2_ from Sr, C, and Sr(N_3_)_2_ in a sealed metal ampoule, an interesting correlation between the amount of azide and the resultant product was seen. The highest amount of N_2_ gas that could be produced from the amount of Sr(N_3_)_2_ used (0.004 g, 0.02 mmol) is very small (0.06 mmol). Assuming that all N_2_ gas produced was present at the reaction temperature (1073 K) and did not react or diffuse into the Na flux, an additional pressure of 0.15 MPa, calculated via the ideal gas equation, was present within the ampoule. However, when a larger amount of azide was used, resulting in a higher amount of N_2_ (0.074 mmol) and thus pressure (maximum of 1.67 MPa), the recently reported compound Sr_4_N[C_2_N][CN_2_] was observed.^2^ This sensitivity of a reaction to the N_2_ pressure was previously observed during the synthesis of Ba_5_[TaN_4_][C_2_N], where, at higher N_2_ pressures, the carbodiimide phase Ba_6_N_5/6_[TaN_4_][CN_2_]_6_ was preferred.^1^ However, the reported synthesis of Sr_4_N[C_2_N][CN_2_] produced this phase both with a Na flux and NaN_3_, but also without Na and azide. This would imply that the N_2_ pressure is not the only factor affecting formation of these compounds.

In order to shed light on the peculiarities of the crystal structure, quantum‐chemical computations were performed. Starting from the experimental structure and placing the nitrogen such that the shorter observed interatomic distance of the [C_2_N]^3−^ units corresponds to a C=N bond (see Figure 1), we obtained an optimised equilibrium structure, which strikingly matches the refined crystal structure (Table S5). The computed interatomic distances are slightly too long by 0.01 Å, but the nearly identical interatomic distances of the C=C and C=N bond, differing by only 0.02 Å, and in particular the very short C=C bond, is confirmed by the calculations. Starting from a structure with inverted [C_2_N]^3−^ anions leads to a significantly less good match with the experimental structure (Table S5) and reduces the computed binding energy by nearly 0.5 eV per unit cell, indicating the absence of orientational disorder of the [C_2_N]^3−^ anion in the experimental structure. We also computed the harmonic force constants and the derived frequencies at the Γ point (Table S6) match the measured Raman frequencies quite well.

Still the question arises, whether the structure observed for the anion is induced by the crystal field or whether it is intrinsic to the [C_2_N]^3−^ anion. To clarify this question, a further set of computations was conducted, this time for the isolated anion. As triply charged anions are not stable in vacuum, the anion was placed inside the cavity of a polarisable, structureless dielectric medium, roughly mimicking a solvated anion. Two screening parameters *ϵ*=2 and *ϵ*=∞, which can be understood as the dielectric constant of the surrounding medium, were used to investigate the influence of the embedding. Irrespective of the value of this parameter, the difference between the C=C and C=N bond lengths increased by 0.06 Å, while the absolute interatomic distances show a clear dependence on *ϵ* (Table S6). It can therefore be immediately concluded that the nearly equal interatomic distances seen in the crystal are an effect of the surrounding counter charges of the Sr^2+^ ions. The vibrational frequencies computed for the isolated ion also show significant shifts compared to those computed for the crystal, in particular for the *v*_3_ mode, which is lowered by more than 200 cm^−1^ for the hypothetical isolated ion (Table S6).

The computed charge distribution for the isolated [C_2_N]^3−^ anion indicates that the excess electrons are located on the terminal atoms. Assuming an electronic structure derived from the isoelectronic CO_2_ molecule or [N_3_]^−^ anion, a formal charge of −2 is expected at the terminal C. In fact, we obtain values between −1.4 *e* and −1.7 *e* for the terminal C, depending on the formalism used to define the partial charges (Table S6). These findings are in line with those from analogous investigations of isolated [C_2_N]^3−^ units in the compound Ba_5_[TaN_4_][C_2_N],^1^ and comparison to results obtained using hybrid functionals and coupled‐cluster methods confirms the predicted structure parameters and charge distributions of the [C_2_N]^3−^ unit (Table S6), thus giving us additional confidence in the accuracy of the density functional computations of the crystal structure.

As discussed above, the crystal field strongly distorts the anion, leading to nearly equal bond lengths and a blue‐shifted *v*_3_ mode. In addition, analysis of the partial charges indicates a strong polarization of the [C_2_N]^3−^ unit, where nearly two electrons are shifted from the carbon atoms towards the nitrogen atom (Table S6).

In summary, the first quasi‐binary acetonitriletriide, Sr_3_[C_2_N]_2,_ has been successfully synthesised and characterised. While not strictly a binary compound, the presence of solely one cationic and one anionic component means this compound is in fact a quasi‐binary salt. The compound crystallises in the space group *P*2_1_/*c* (No. 14), where all [C_2_N]^3−^ units are located in bidisphenoids of Sr cations. This structural motif is also seen in the structurally similar compound Sr_4_N[C_2_N][CN_2_], which presents disorder of both three‐atomic anions. The formation of the acetonitriletriide ion is sensitive to N_2_ pressure. At lower N_2_ pressures, the title compound is obtained; however, at higher N_2_ pressures Sr_4_N[C_2_N][CN_2_] is produced. This work provides a synthetic route to the as yet unknown family of pseudonitrides, which like nitrides and carbodiimides could exhibit attractive physical and chemical properties, when combined with suitable cations, for example from the group of transition metals.

## Conflict of interest

The authors declare no conflict of interest.

## Supporting information

As a service to our authors and readers, this journal provides supporting information supplied by the authors. Such materials are peer reviewed and may be re‐organized for online delivery, but are not copy‐edited or typeset. Technical support issues arising from supporting information (other than missing files) should be addressed to the authors.

Supplementary
