# Supplementary material for: The Quasi‐Binary Acetonitriletriide Sr3[C2N]2
Source: Angew Chem Int Ed Engl. 2019 Nov 19;59(1):339–42. doi: 10.1002/anie.201912831 (PMC6973148; doi:10.1002/anie.201912831)
Supplement: Supplementary file 1 — Supplementary [file ANIE-59-339-s001.pdf]

Angewandte Chemie
Eine Zeitschrift der Gesellschaft Deutscher Chemiker

Supporting Information

The Quasi-Binary Acetonitriletriide $Sr_3[C_2N]_2$

*William P. Clark, Andreas Köhn, and Rainer Niewa**

anie_201912831_sm_miscellaneous_information.pdf

Table of Contents

1. Experimental procedure 3
2. Crystal structure of $Sr_3[C_2N]_2$ 4
3. Elemental analysis 5
4. Computational results 6
References 7
Author Contributions 7

1. Experimental procedure

Inside an Ar filled glovebox, a mixture of Sr (0.092 g, 1.05 mmol), C (0.005 g, 0.42 mmol) and $Sr(N_3)_2$ (0.004 g, 0.02 mmol) was filled into a Ni ampoule with elemental Na (0.130 g) as a fluxing agent. Using an arc welder, integrated into the glovebox, the ampoule was sealed and inserted into a fused silica tube, which was then transferred to a tube furnace. Under Ar flow the sample was heated to 1073 K, at 100 K/h, and held for 6 hours, before being allowed to cool to room temperature at a very slow rate of 1 K/h. The ampoule was then opened in the glovebox and the Na flux was removed by extracting with $NH_{3(l)}$ using a Tensi-Eudiometer.[1] The resulting sample contained single crystals, which were near colourless, with a hint of green, and unreacted Sr.

Diffraction experiments were carried out using a single crystal X-ray diffraction on a Kappa-CCD Bruker-Nonius single crystal diffractometer (Mo-K_α radiation, λ = 0.71073 Å) and analysed with the ShelX software package.[2] Further details on the crystal structure investigation may be obtained from the Fachinformationszentrum Karlsruhe, 76344 Eggenstein-Leopoldshafen, Germany (fax: (+49)7247-808-666; e-mail: crysdata@fiz-karlsruhe.de), on quoting the depository number CSD-1939396.

Raman spectroscopy was measured, between 0 and 2000 cm^{-1}, with an Olympus confocal polarization microscope BX51, using a green laser (λ = 532 nm). A single near colourless crystal of the product was inserted and sealed in a glass capillary, to prevent the sample reacting with air.

Elemental analysis was conducted by energy dispersive spectroscopy, using a Cameca SX-100 electron microscope. Sample preparation involved sputtering the single crystal sample with a thin coat of C.

2. Crystal structure of $Sr_3[C_2N]_2$

Table S1. Single crystal structural refinement of $Sr_3[C_2N]_2$.

Crystal System	Monoclinic
Space Group	$P2_1/c$ (No. 14)
Z	2
a/Å	4.0745(1)
b/Å	10.7254(5)
c/Å	7.0254(3)
β/°	102.700(2)
ρ_{calc}/gcm^{-3}	3.758
Volume V/Å^3	299.50
Measurement Temperature/K	293(2)
Index range	$-6 \leq h \leq 5$<br>$-13 \leq k \leq 13$<br>$-4 \leq l \leq 9$
Max. 2θ /deg	55.03
F(000)	304.0
μ/mm^{-1}	26.50
Observed reflections	6116
Unique reflections	690
Refined parameters	45
R_σ	0.0209
R_1/wR_2	0.0305/0.0955
$GooF$	1.153
Remaining electron density (max/min)/Å^{-3}	1.44/–0.83
BASF	0.124(4)
Twin matrix	1 0 0 0 −1 0 −¾ 0 1

Table S2. Fractional atomic positions, occupational and isotropic displacement parameters (Å^2) for the structure refinement of $Sr_3[C_2N]_2$.

	Wyckoff Position	x/a	y/b	z/c	U_{eq}
Sr(1)	2a	0	0	0	0.0140(3)
Sr(2)	4e	0.6722(1)	0.15468(5)	0.42524(8)	0.0141(3)
N(1)	4e	0.086(1)	0.2106(5)	0.1917(8)	0.018(1)
C(1)	4e	0.231(1)	0.1831(6)	0.7149(8)	0.012(1)
C(2)	4e	0.386(2)	0.0776(6)	0.7355(9)	0.018(1)

Table S3. Anisotropic displacement parameters (Å^2) from the structure refinement of $Sr_3[C_2N]_2$.

	U_{11}	U_{22}	U_{33}	U_{23}	U_{13}	U_{12}	U_{eq}
Sr(1)	0.0134(4)	0.0158(5)	0.0130(4)	−0.0016(3)	0.0034(3)	−0.0007(3)	0.0140(3)
Sr(2)	0.0128(4)	0.0168(4)	0.0122(4)	−0.0003(2)	0.0017(2)	−0.0010(2)	0.0141(3)
N(1)	0.014(2)	0.020(3)	0.018(3)	−0.002(2)	0.002(2)	−0.002(2)	0.018(1)
C(1)	0.011(3)	0.015(3)	0.012(3)	−0.000(2)	0.005(2)	−0.007(2)	0.012(1)
C(2)	0.021(3)	0.015(3)	0.018(3)	−0.001(2)	0.008(2)	−0.002(2)	0.018(1)

3. Elemental analysis

To identify the elemental components of the sample energy dispersive spectroscopy was used. Since a thin coating of C on the surface of the sample is needed for the measurement, detection of C within the sample was not possible. Also, during the mounting of the samples onto the apparatus, the sample is exposed to air for a short period. Since the sample is sensitive to air and moisture, the accurate determination of N content was also not possible. These limitations meant that only qualitative information about the cations present could be determined. The results showed that Sr is the only other detectable element present, except for C, N and O (Table S4). This leads to the conclusion that the $[C_2N]^{3-}$ units are contained in a mono-cationic environment.

Table S4. Relative values from energy dispersive spectroscopy for $Sr_3[C_2N]_2$.

	w(Sr)/%	*w*(C)/%	*w*(N)/%	*w*(O)/%
1	71.30±0.30	4.56±0.09	4.24±0.32	19.91±0.18
2	71.24±0.30	5.33±0.09	3.89±0.32	19.53±0.14

4. Computational results

Solid state computations have been carried out with the VASP program.[3] Density functional theory (DFT) using the Perdew-Burke-Ernzerhof[4] (PBE) functional and the projector-augmented wave (PAW)[5,6] method was employed. The plane wave cut-off was 400 eV and k-point integration used a 4x4x4 Monkhorst-Pack[7] grid.

The molecular DFT and coupled-cluster computations were done with the Molpro program package.[8,9] The PBE functional and its hybrid variant[10,11] PBE0 were employed, as well as atom-centred def2-QZVPP basis sets.[11] The highly charged anion was stabilized by placing it inside a cavity of a structureless polarizable medium (conductor-like screening model, COSMO).[12] Partial charges were determined by a projection approach (intrinsic atomic orbitals, IAO),[13] natural atomic orbitals were also tried,[14] leading to qualitatively the same results within 10%. In addition, a Bader charge analysis was carried out, using the program of the Henkelman group (https://theory.cm.utexas.edu/henkelman/code/bader/).[15] This charge analysis was also available for the solid state computations. The results of the Bader analysis differ from those of the IAO approach by approximately 0.5 *e* being assigned to the N atom instead of the C atoms.

Table S5. Experimental and computed (DFT using the PBE functional, plane wave cut-off 400 eV) structure parameters. In the 'inverted' structure, the $[C_2N]^{3-}$ anions point into the opposite direction.

	V/Å^3	a/Å	b/Å	c/Å	β/°	d(CC)/Å	d(CN)/Å
Exp.	299.5	4.0450	10.7254	7.0254	102.7	1.291(9)	1.271(9)
Calc.	295.2	4.0458	10.7007	6.9799	102.4	1.301	1.281
Calc. (inv.)	299.2	4.0046	10.7731	7.0397	99.9	1.300	1.288

Table S6. Computed properties of the $[C_2N]^{3-}$ anion. The computations use COSMO embedding (effective dielectric constant ε) and def2-QZVPP basis sets (only the anion is considered). The partial charges have been obtained by a grid-based Bader analysis and by the intrinsic atomic orbital (IAO) approach (numbers in parentheses).

	d(CC)/Å	d(CN)/Å	δ/cm^{-1}	ν_1 / cm^{-1}	ν_3 / cm^{-1}	$q(C_1)$	$q(C_2)$	q(N)
Isolated:								
PBE (ε = 2)	1.347	1.285	580	1065	1597	–1.41 (–1.67)	+0.09 (–0.13)	–1.66 (–1.20)
PBE (ε = ∞)	1.331	1.269	581	1122	1622	–1.41 (–1.71)	+0.04 (–0.13)	–1.62 (–1.16)
PBE0 (ε = 2)	1.334	1.270	618	1125	1644	–1.39 (–1.69)	+0.12 (–0.09)	–1.72 (–1.22)
PBE0 (ε = ∞)	1.318	1.255	619	1181	1653	–1.39 (–1.73)	+0.13 (–0.08)	–1.72 (–1.18)
CCSD(T) (ε = 2)[a]	1.341	1.286	—	—	—	—	—	—
CCSD(T) (ε = ∞)[a]	1.328	1.271	—	—	—	—	—	—
Crystal:								
PBE	1.301	1.281	663<br>663<br>662<br>659<br>620<br>619<br>616<br>616	1191<br>1187<br>1186<br>1184	1897<br>1842<br>1827<br>1823	–0.43	+0.99	–2.80
Exp.	1.291(9)	1.271(9)	508<br>599<br>651	1073<br>1139<br>1146	1715<br>1744<br>1826			

[a] COSMO reaction field computed for mean-field potential only. Frequency calculations were not possible at this level due to too strong numerical noise from this approach. [b] Eigenvalues of the dynamical matrix at the Γ point.

References

[1] G. Hüttig, *Z. Anorg. Allg. Chem.* **1920**, *109*, 162–173.

[2] a) *G. M. Sheldrick*, Program SHELX-97, **1997**; b) *Stoe & Cie GmbH*, Program X-RED 32, **2005**; c) *Stoe & Cie GmbH*, Program X-STEP 32, **2000**; d) *Stoe & Cie GmbH*, Program X-SHAPE 32, **1999**.

[3] G. Kresse et al., VASP, version 5.4.1, ab-initio simulation package, **2016**, For more information, see https://www.vasp.at.

[4] J. P. Perdew, K. Burke, M. Ernzerhof, *Phys. Rev. Lett.* **1996**, *77*, 3865–3868.

[5] P. E. Blöchl, *Phys. Rev. B* **1994**, *50*, 17953–17979.

[6] G. Kresse, D. Joubert, *Phy. Rev. B* **1999**, *59*, 11–19.

[7] J. D. Pack, H. J. Monkhorst, *Phys. Rev. B* **1977**, *16*, 1748–1749.

[8] H.-J. Werner, P. J. Knowles et al., MOLPRO, version 2018.1, a package of ab initio programs, **2018**, For more information, see https://www.molpro.net.

[9] H.-J. Werner, P. J. Knowles, G. Knizia, F. R. Manby, M. Schütz, *Wiley Interdiscip. Rev. Comput. Mol. Sci.* **2012**, *2*, 242–253.

[10] C. Adamo, V. Barone, *J. Chem. Phys.* **2001**, *110*, 6158–6170.

[11] F. Weigend, R. Ahlrichs, *Phys. Chem. Chem. Phys.* **2005**, *7*, 3297–3305.

[12] A. Klamt, G. Schüürmann, *J. Chem. Soc. Perkin Trans.* **1993**, *2*, 799–805.

[13] G. Knizia, *J. Chem. Theory Comput.* **2013**, *9*, 4834–4843.

[14] A. E. Reed, R. B. Weinstock, F. Weinhold, *J. Chem. Phys* **1985**, *83*, 735–746.

[15] W. Tang, E. Sanville, G. Henkelman, *J. Phys. Condens. Matter* **2009**, *21*, 084204.

Author Contributions

W.P.C performed the experiments and data analysis. A.K. performed the calculations. R.N. supervised the project and took part in the data analysis and interpretation. W.P.C. and R.N. prepared the manuscript.
